# HDAC6 Inhibitor Blocks Amyloid Beta-Induced Impairment of Mitochondrial Transport in Hippocampal Neurons

**DOI:** 10.1371/journal.pone.0042983

**Published:** 2012-08-22

**Authors:** Chaeyoung Kim, Heesun Choi, Eun Sun Jung, Wonik Lee, Soojung Oh, Noo Li Jeon, Inhee Mook-Jung

**Affiliations:** 1 Department of Biochemistry and Biomedical Sciences, Seoul National University College of Medicine, Seoul, Korea; 2 School of Mechanical and Aerospace Engineering, Seoul National University, Seoul, Korea; 3 World Class University (WCU) Program of Multiscale Design, School of Mechanical and Aerospace Engineering, Seoul National University, Seoul, Korea; Federal University of Rio de Janeiro, Brazil

## Abstract

Even though the disruption of axonal transport is an important pathophysiological factor in neurodegenerative diseases including Alzheimer's disease (AD), the relationship between disruption of axonal transport and pathogenesis of AD is poorly understood. Considering that α-tubulin acetylation is an important factor in axonal transport and that Aβ impairs mitochondrial axonal transport, we manipulated the level of α-tubulin acetylation in hippocampal neurons with Aβ cultured in a microfluidic system and examined its effect on mitochondrial axonal transport. We found that inhibiting histone deacetylase 6 (HDAC6), which deacetylates α-tubulin, significantly restored the velocity and motility of the mitochondria in both anterograde and retrograde axonal transports, which would be otherwise compromised by Aβ. The inhibition of HDAC6 also recovered the length of the mitochondria that had been shortened by Aβ to a normal level. These results suggest that the inhibition of HDAC6 significantly rescues hippocampal neurons from Aβ-induced impairment of mitochondrial axonal transport as well as mitochondrial length. The results presented in this paper identify HDAC6 as an important regulator of mitochondrial transport as well as elongation and, thus, a potential target whose pharmacological inhibition contributes to improving mitochondrial dynamics in Aβ treated neurons.

## Introduction

Neurons have extensive processes and asymmetrical organization. The communication between the cell body and the nerve terminal is critical for neuronal functions, which involves microtubule (MT) based axonal transport. MTs are dynamically assembled polymers of α- and β-tubulin [1]. Tubulin undergoes various post-translational modifications (PTMs), including acetylation, tyrosination, and phosphorylation [2], [3]. PTMs of tubulin regulate not only the interaction between MTs and MT-associated proteins (MAP), but also the stability of the microtubule, contributing to controlling axon guidance, synapse formation, and neuronal transport [2], [3], [4]. Acetylation of α-tubulin plays a positive role in axonal transport in mammals by increasing microtubule stability [3], [5], [6]. Histone deacetylase 6 (HDAC6) is a unique cytosolic enzyme that mediates the deacetylation of α-tubulin, which involves two functional deacetylase domains and a zinc finger motif [7]. The level of acetylated α-tubulin is decreased [9], [10] as the level of HDAC6 is increased in the AD patients' brains [11]. Since impaired axonal transport is an important pathophysiological factor in AD [8], HDAC6 may play a role in the disruption of axonal transport in AD pathogenesis. Amyloid beta (Aβ), the cleavage product from amyloid precursor protein (APP), is one of the causative factors of AD pathogenesis. Aβ interrupts vesicular and axonal transport by inducing alteration in microtubule stability and intracellular signaling pathways [12], [13], [14]. Aβ also causes synaptic degeneration and loss through the disruption of axonal transport [12], [15], which leads to impaired trafficking of the mitochondria and neurotransmitters necessary for synaptic function and neuronal viability [8], [10], [16].

Mitochondria can be delivered along the axon in association with microtubules, which is important for supplying energy required to maintain neuronal functions. During axonal transport, mitochondria are associated with several motor proteins, such as kinesin for anterograde transport and dynein for retrograde transport [1], [2]. Adaptor proteins, such as Miro (Miro1 and Miro2 in mammals) and Milton (OIP106 and GRIF1 in mammals), are connected to mitochondria through kinesin [17], [18]. Although impaired axonal transport of mitochondria has been reported in the presence of Aβ [12], the precise mechanism for this Aβ-induced impairment remains unclear. In the present study, we attempted to elucidate the mechanism that links the acetylation of α-tubulin and Aβ-induced impairment of mitochondrial transport in hippocampal neurons cultured in a microfluidic system. To increase α-tubulin acetylation, we used the Tubastatin A (TBA) as the HDAC6 inhibitor. Mitochondrial axonal transport was analyzed by measuring the velocity, motility and length of mitochondria. We found that pharmacological inhibition of HDAC6 significantly restored the compromised velocity and motility of the mitochondria of Aβ hippocampal neurons to a normal level in both anterograde and retrograde axonal transports. The inhibition of HDAC6 also recovered the length of mitochondria that had been shortened by Aβ. These results show that the inhibition of HDAC6 rescued neuronal cells from Aβ-induced impairment of mitochondrial axonal transport as well as mitochondrial length, identifying HDAC6 as a potential therapeutic target to modulate AD pathogenesis.

## Results

### Decrease in the acetylation of α-tubulin in the brains of 5XFAD mice

**Figure 1:**
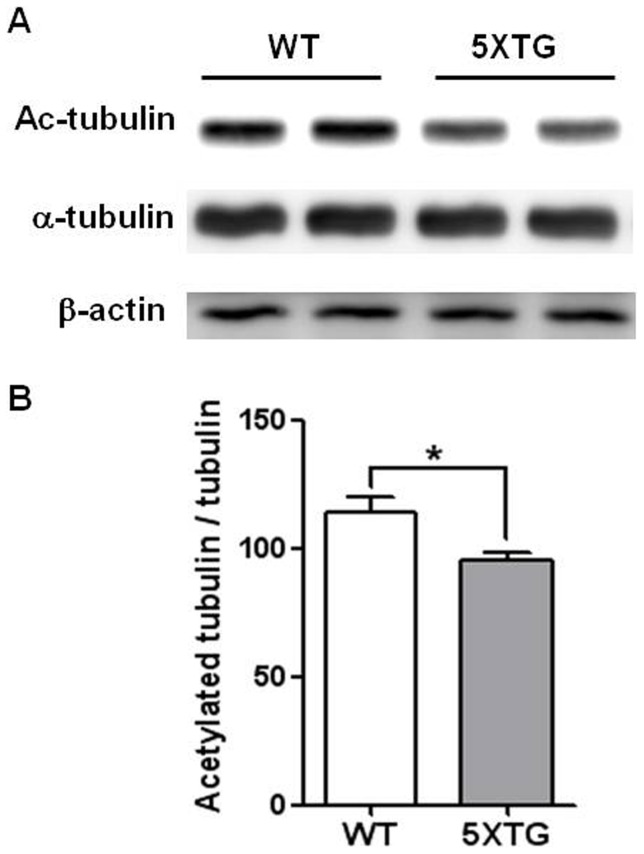
Reduction of acetylated α-tubulin in 5XFAD. (A) Western blot of acetylated α-tubulin in the brains of both wild type (WT) and 5XFAD mice. Brain extracts were prepared from frontal cortex of 13-month-old mice. Actin is a loading control. (B) Quantitation of the acetylated α-tubulin normalized by total α-tubulin is shown as means ± SEM (WT n = 4, 5XFAD n = 3, *P<0.05).

The reduced level of acetylated α-tubulin [9], [10] correlates to the increased level of HDAC6 in the AD patients' brains [11]. To determine the role of acetylation of α-tubulin in AD, we examined the level of acetylated α-tubulin in the brains of 5XFAD mice, an AD animal model. Western blot analysis showed that the level of acetylated α-tubulin in the 5XFAD mice was significantly lower than that of wild type mice (WT: 1.145±0.059, 5XFAD: 0.96±0.026, Fig. 1A and 1B, *p<0.05), whereas no difference was observed in the amount of total α-tubulin. We, therefore, conclude that acetylated α-tubulin level is decreased in the brains of 5XFAD mice.

### Regulation of the acetylation of α-tubulin by Aβ and HDAC6 inhibitor

**Figure 2:**
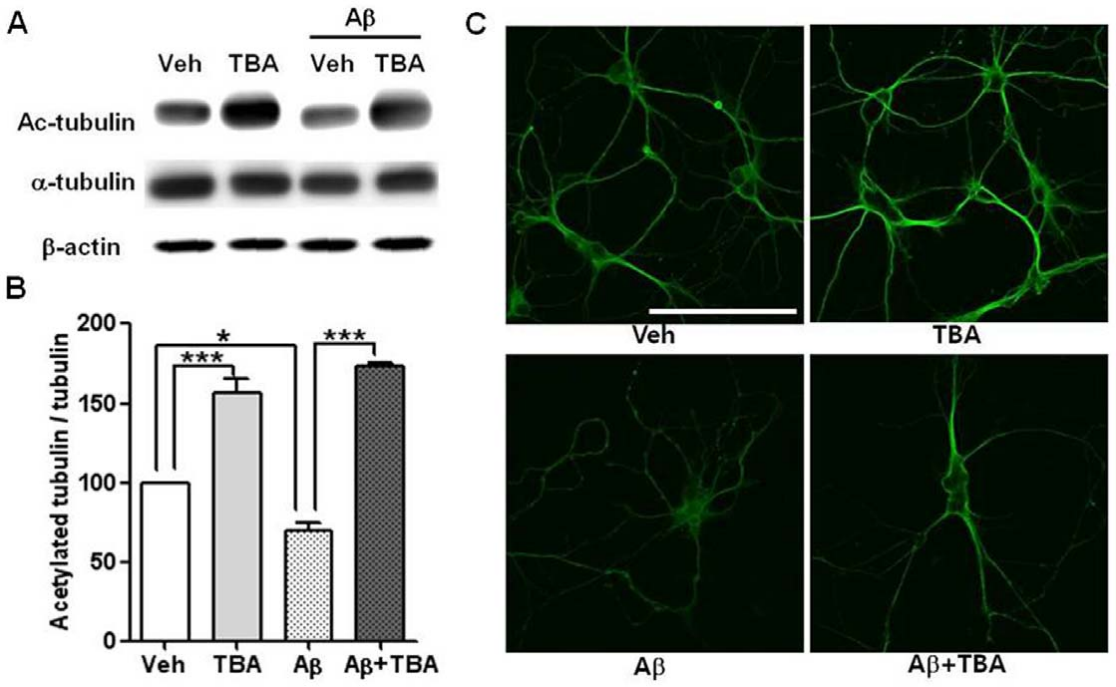
Modulation of acetylated α-tubulin by Aβ and TBA. (A) Western blot of acetylated α-tubulin in the rat hippocampal neurons. After being pretreated with Aβ (2 µM) for 24 hrs, cells were treated with TBA (5 µM) for 3 hrs and lysed with RIPA buffer. Actin is a loading control. (B) Quantitation of the acetylated α-tubulin normalized by total α-tubulin is shown as means ± SEM. Data were acquired from 4 independent experiments (*P<0.05, ***P<0.001). (C) Immunocytochemistry of acetylated α-tubulin in hippocampal neurons. Anti-acetylated α-tubulin antibody detects α-tubulin only when acetylated at Lys 40 (Scale bar = 100 µm).

It has been reported that Aβ alters the level of acetylated α-tubulin in primary neuronal cultures and cell lines [10] whereas the HDAC6 inhibitor, TBA, promotes the acetylation of α-tubulin [19], [20], [21]. To examine the role of Aβ in reducing acetylated α-tubulin, primary hippocampal neurons were characterized. Consistent with previous studies [10], [22], the level of acetylated α-tubulin was significantly decreased by the Aβ treatment (Fig. 2), as shown by Western blot analysis (Fig. 2A and 2B, *p<0.05, ***P<0.001) as well as immunocytochemistry (Fig. 2C). Western blot analysis (Fig. 2A and 2B, *p<0.05, ***P<0.001), together with immunocytochemistry (Fig. 2C), revealed that TBA treatment in the presence of Aβ restored Aβ-induced reduction in the level of acetylated α-tubulin level, compared to Aβ treatment alone. This indicates that TBA increased α-tubulin acetylation even in the presence of Aβ.

### The effect of Aβ and TBA on the velocity and motility in mitochondrial axonal transport

**Figure 3:**
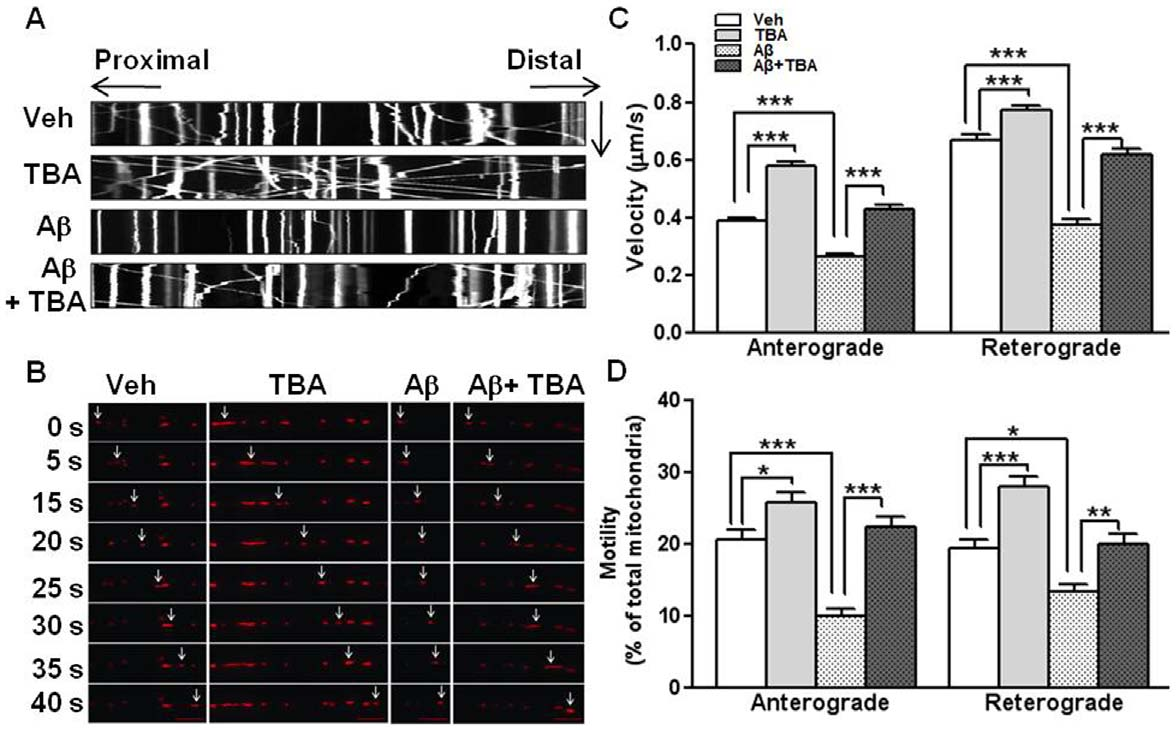
Regulation of mitochondrial transport by Aβ and TBA. (A) Representative kymographs of mitochondrial movement. Hippocampal neurons from rat (E18) were plated with densities of 6×10^4^ cells in the somal side of the microfluidic chamber. Cells were transfected with pDsRed2-Mito after 7 days of culture. After being pretreated with Aβ (2 µM) for 24 hrs, cells were treated with TBA (5 µM) for 3 hrs. Images were acquired every 1 sec for 2 min at microgrooves. X axis of kymograph is axonal length (152.7 µm). Proximal to distal indicates the soma to axon terminal direction. Mitochondria which move from proximal to distal region show anterograde movement. Y axis is the time that mitochondria have moved (2 min). (B) Pictures of motile mitochondria for each group were shown every 5 sec (Scale bars = 10 µm). Arrows indicate motile mitochondria. (C) Average velocity of motile mitochondria. Anterograde and retrograde velocity were analyzed separately. (D) Motility of mitochondria. Motility stands for percentage of motile mitochondria over total mitochondria. Anterograde and retrograde motility were analyzed separately. Data were acquired from 4 independent experiments (Veh n = 41, TBA n = 44, Aβ n = 42, Aβ+TBA n = 43, *P<0.05, **P<0.01, ***P<0.001).

Because it has been reported that mitochondrial transport along the microtubule was disrupted in the presence of Aβ [12], the effect of TBA in the presence of Aβ on mitochondrial transport was examined with primary hippocampal neuron cultures in microfluidic system, which could isolate neuronal compartments between the soma and axon. To visualize mitochondrial movement, pDsRed2-Mito construct was transfected into the DIV 7 neurons, and live cell imaging was performed after the neurons were treated with Aβ and/or TBA. 24 hrs after transfection, the neurons were pretreated with 2 µM Aβ for 24 hrs and then treated with 5 µM TBA for 3 hrs in both somal and axonal sides. Images were taken every sec for 2 mins. To analyze the direction of velocity and motility of mitochondria, we used z-projection and multiple Kymograph plugins functions of the ImageJ program. We found that Aβ greatly reduced mitochondrial movement, and TBA recovered the reduced mitochondrial movement in the presence of Aβ, as shown by the kymographs (Fig. 3A, B, and Movie S1, S2, S3, S4). In Aβ treated neurons, we observed the decreased velocity in both anterograde and retrograde movements of mitochondria. TBA treatment with Aβ induced a recovery of the reduced velocity of mitochondria by Aβ (Fig. 3C, ***p<0.001). The results for motility were consistent with those for velocity (Fig. 3D, *p<0.05, **p<0.01, ***p<0.001). In this experiment, the ration of motile mitochondria over total mitochondria was calculated to present the motility of mitochondria. Consistent with the data for mitochondrial velocity (Fig. 3C), mitochondrial motility was also decreased by Aβ and recovered by TBA treatment in both directions (Fig. 3D). In the group treated with both Aβ with TBA, the defects caused by Aβ were all rescued by TBA treatment (Fig. 3), suggesting that acetylated α-tubulin regulates mitochondrial transport even in the presence of Aβ.

### The effect of Aβ and TBA on mitochondrial length in axon

**Figure 4:**
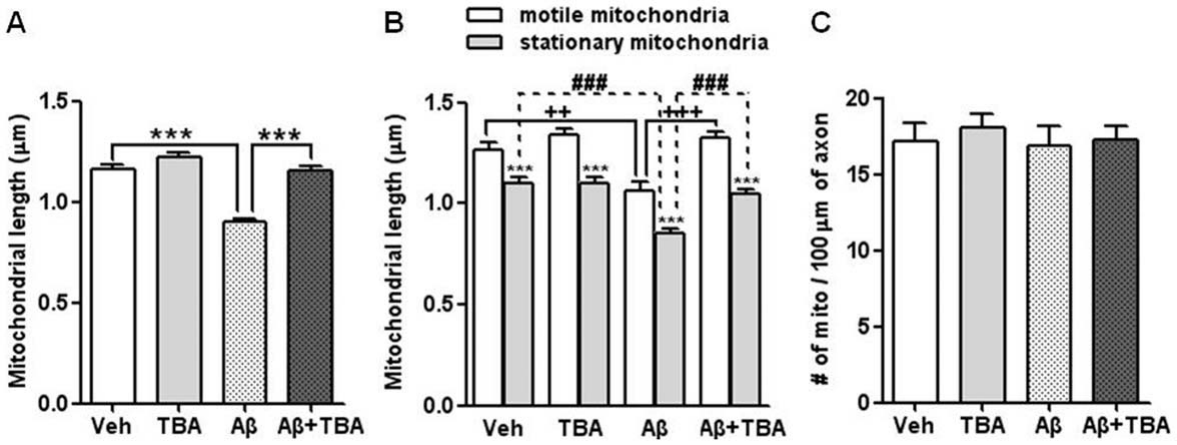
Alteration of mitochondrial length by Aβ and TBA. (A) Average length of total mitochondria, including both motile (anterograde, retrograde transported) and stationary mitochondria. (B) Average length of motile and stationary mitochondria. ***P<0.001 significance of stationary mitochondria vs. motile mitochondria; ++P<0.01, +++P<0.001 among motile mitochondria; ###P<0.001 among stationary mitochondria. (C) The average number of mitochondria per 100 µm of axon. Data were obtained from 4 independent experiments (Veh n = 35, TBA n = 42, Aβ n = 38, Aβ & TBA n = 38, *P<0.05, **P<0.01, ***P<0.001).

It has been shown that the motor protein dynein is involved in mitochondrial morphology [23], [24] and that pharmacological inhibition of HDAC affects mitochondrial elongation [25]. To test the hypothesis that the acetylation of α-tubulin affects mitochondrial length in hippocampal neurons, we determined the effect of HDAC6 inhibition on mitochondrial length by treating the cells with TBA in the absence or presence of Aβ and measured mitochondrial length. In parallel, to compare the length and motility of mitochondria, we separately determined the length of stationary and motile mitochondria. Overall, mitochondrial length was decreased by Aβ and increased in the group co-treated with Aβ and TBA (Fig. 4A; Veh: 1.168±0.02, Aβ: 0.904±0.02, TBA: 1.228±0.02, Aβ & TBA: 1.159±0.02, ***p<0.001). Although both motile and stationary mitochondria showed a similar pattern as shown in Fig. 4B, the length of stationary mitochondria was shorter than that of motile mitochondria (Fig. 4B, **p<0.01, ***p<0.001), suggesting that longer mitochondria have higher motility than shorter ones. This demonstrates that mitochondrial length is related to mitochondrial transport and that TBA treatment affects both mitochondrial length and motility in neurons. However, there was no significant difference among the four groups in the number of mitochondria per 100 µm of axon (Fig. 4C). These findings suggest that Aβ and HDAC6 are important in mitochondrial length and axonal transport.

## Discussion

Impaired axonal transport and mitochondrial dysfunctions occur in the early stages of AD [8], [15]. These changes are induced by Aβ which forms amyloid plaques, one of the major hallmarks of AD [26], [27], [28], [29]. Increased GSK3β activity induces hyperphosphorylation of tau, resulting in the formation of neurofibrillary tangles (NFT) and/or an increase in Aβ generation[30]. HDAC6 activity, as a substrate of GSK3β, is increased by GSK3β activation [31], suggesting a decrease in both the level of α-tubulin acetylation and axonal transport. To modulate HDAC6 activity, we used TBA as a potent HDAC6 inhibitor, which is more selective than other HDAC6 inhibitors, such as TSA and tubacin [21], [32]. The specificity of TBA to HDAC6 was confirmed in previous studies by homology modeling and enzyme inhibition experiments using 11 HDAC isozymes [21], [32]. TBA affects the acetylation of cytosolic proteins like α-tubulin but not histones [21]. The knockdown of HDAC6 by siRNA resulted in an increased level of acetylated α-tubulin, consistent with the results with the HDAC6 inhibitor [33]. It has been reported that TBA treatment increases not only axonal transport and the number of moving mitochondria but also the level of acetylated α-tubulin in neuropathies of a Charcot-Marie-Tooth model that have been induced by mutant heat-shock protein gene (HSPB1) [32]. Given the results from the current and previous studies, it is increasingly clear that TBA specifically inhibits HDAC6 and, thus, affects the acetylation of α-tubulin and axonal transport of cellular cargoes, such as mitochondria. Motor proteins, such as kinesin for anterograde transport and dynein for retrograde transport, are involved in mitochondrial transport [34]. Adaptor proteins, such as milton, syntabulin, miro (for kinesin), and dynactin (for dynein), are also required [17], [18]. Axonal transport is regulated by the interaction between adaptor and motor proteins as well as their stability is important in axonal transport [17], [18], [34]. It has been also known that α-tubulin acetylation at Lys 40 increases the interaction between kinesin and the microtubule, resulting in improved transport of cargo proteins [3]. Dompierre et al. have reported that MT acetylation increases both anterograde and retrograde transport, which was attributed to the increased interaction of both kinesin family motor protein 5 (KIF5) and dynein with MTs [35]. However, the mechanism by which acetylation of α-tubulin regulates mitochondrial transport under pathological conditions, such as the accumulation of Aβ, is unclear. Du et al. [15] have reported that mitochondrial motility was significantly reduced in both directions (anterograde and retrograde) in neurons treated with 200 nM Aβ oligomer. By contrast, Calkins et al. [12] found that the treatment with 20 µM Aβ_25–35_ oligomer significantly reduced the motility of anterograde mitochondrial transport but not the velocity in mitochondrial transport in both directions. Moreover, Wang and colleagues [36] have shown that the motility of mitochondria was decreased in both directions. In the current study, we showed that the treatment of neurons with 2 µM Aβ significantly reduced the motility and velocity in mitochondrial transport in both directions. The difference in the level and direction of mitochondrial motility and velocity between this and other studies may be relevant to the form or concentration of Aβ. Our AFM assay showed that Aβ used in this experiment is mostly in oligomer form even though other types of Aβ are present in the mixture (see the figure in File S1). Although the exact mechanism underlying TBA-mediated enhancement of axonal transport is not fully studied, we suggest that TBA promotes the binding of motor proteins to MTs, contributing to the restoration of mitochondrial transport that has been impaired by Aβ. The size and structure of mitochondria are dynamically regulated by mitochondrial fission and fusion [37], [38]. Aβ activates two mitochondrial fission proteins, Fis1 and Drp1, leading to excessive fragmentation of mitochondria that may disrupt mitochondrial transport [12]. Misko et al. (2010) also reported that mitofusin 2, an outer mitochondrial membrane protein involved in regulating mitochondrial dynamics, is directly involved in mitochondrial axonal transport [39]. Our results (Fig. 4B) show that motile mitochondria are longer than stationary ones. Thus, it is likely that the change in mitochondrial length is coupled with mitochondrial transport. Therefore, we tentatively anticipate that HADC6 is more likely to regulate the mitochondrial dynamics. An outstanding issue that remains to be further investigated is how HDAC6 inhibitors affect the interaction between an adaptor protein and MTs and the functions of the mitochondria. In addition, it remains to be further examined whether mitochondrial elongation directly leads to increased mitochondrial transport. Since Aβ has been shown to reduce ATP production in neurons [40], we examined whether TBA-treated neurons are recovered from Aβ-induced impairment in ATP generation. When ATP production was measured in this system, Aβ treatment reduced ATP production significantly, but co-treatment of Aβ with TBA did not rescue Aβ-induced reduction of ATP production (data not shown), suggesting that ATP production is not associated with axonal transport and morphology of the mitochondria in cells treated with TBA. Regarding the relationship of changes in membrane potential to mitochondria and axonal transport, Miller and Sheetz (2004) reported that mitochondria with high membrane potential moved toward the axon terminus, whereas mitochondria with low membrane potential moved toward the soma [41], suggesting that membrane potential is another factor that influences mitochondrial axonal transport. Overall, this study showed that Aβ impaired bidirectional mitochondrial transport, which was subsequently rescued by the HDAC6 inhibitor. Furthermore, we observed that the changes in both acetylation of α-tubulin and mitochondrial elongation correlate to the alteration of mitochondrial transport in cells treated with Aβ and/or an HDAC6 inhibitor. Therefore, these results suggest that an HDAC6 inhibitor may be a potential therapeutic target for the treatment of AD.

## Materials and Methods

### Animals

Female Sprague-Dawley (SD) rats whose embryos (E18) were used for primary hippocampal neuronal culture were purchased from KOATECH (Gyeonggido, Korea). The brain tissues of 13-month-old 5XFAD mice (Tg6799; B6SJL-Tg (APPSwFlLon, PSEN* M146L*L286V) 6799Vas/J, stock number 006554, Jackson Labs, Bar Harbor, ME) overexpressed three mutations (Swedish, Florida, and London) of human APP 695 and two mutations (M146L and L286V) of human PS1 under transcriptional control of the murine Thy-1-promoter and B6SJL wild type (WT) mice were used for western blot analysis. Animals were treated and maintained as per the Helsinki Treaty, the Principles of Laboratory Animal Care (NIH publication No. 85-23, revised 1985), and the Animal Care and Use Guidelines of Seoul National University, Seoul, Korea. All procedures for animal experiments were approved by the Ethics Review Committee for Animal Experimentation in Seoul National University (approval number, SNU060519-5, IACUC).

### DNA construct and reagents

pDsRed2-Mito (Clontech, Mountain View, CA) is a mammalian expression vector that encodes a red fluorescent protein and the mitochondrial targeting sequence, resulting in neuronal mitochondria appearing as a red fluorescence. Acetylated α-tubulin and β-actin antibodies were purchased from Sigma-Aldrich (St. Louis, MO) and α-tubulin antibodies were from abm (Applied Biological Materials Inc., Richmond, BC). Alexa Fluor 488 donkey anti-mouse IgG was used in immunofluorescence analysis. Tubastatin A (TBA; BioVision, Mountain View, California) and Aβ_1–42_ peptides (American peptide, Sunnyvale, CA) were also used.

### Cell cultures and transfection

Primary hippocampal neurons were obtained from the brain tissue of SD rat embryos (E18). Preparation of primary hippocampal neurons was followed in previous reports with minor modifications [15], [42]. Briefly, brain tissues were isolated in ice-cold Hank's Balanced Salt Solution (HBSS; WelGENE, Daegu, Korea) and digested with 0.5% trypsin (using 2.5% trypsin; Sigma, St. Louis, MO). Cells were dissociated in Neurobasal/B27 medium (Invitrogen, Carlsbad, California) with 0.1 mg/ml penicilin/streptomycin (Sigma, St. Louis, MO) and plated onto poly-D-lysine (Sigma, St. Louis, MO) coated plates and microfluidic chambers. Half of the culture medium was replaced by fresh medium every 2 days for plates and every day for microfluidic chambers. For labeling mitochondria, pDsRed2-Mito was transfected into neurons at 7 days in vitro (DIV) using Lipofectamine 2000 (Invitrogen, Carlsbad, California). After being pretreated with Aβ_1–42_ peptides (2 µM) at DIV 8 for 24 hrs, cells were treated with TBA (5 µM) for 3 hrs [21].

### Microfluidic chamber system

The microfluidic chambers for separating axons were fabricated as described in the reference [43]. After assembling the chambers, the chambers were coated by poly-D-lysine. Cells were loaded in only one compartment of the chamber, the somal compartment. As neurites were growing up, the other compartment was filled with axon which we called the axonal compartment. All chambers have a microgroove length of 900 µm and a width of 8 µm.

### Live cell imaging and image analysis

Living cells were imaged using Olympus IX81 microscope (Tokyo, Japan) equipped with a Cool SNAP HQ2 CCD camera (Photometrics, Ltd., Tucson, AZ 85706), controlled by MetaMorph Software (Universal Imaging, PA, USA). During imaging, cells were maintained in an incubating chamber at 37°C and supply atmosphere of 5% CO_2_/95% air (Live cell instrument, Seoul, Korea). Time-lapse image recordings were acquired at an exposure time of 500 ms, 1 sec interval and duration up to 2 min. Live cell images were obtained from before and after TBA treatment and focused on axons in microgroove. Movies were processed using MetaMorph. Quantitative analysis of mitochondrial velocity and motility was performed manually using ImageJ (rsb.info.nih.gov, by W. Rasband) installed multiple kymograph plugins (by J. Rietdorf and A. Seitz) at each axon. In order to analyze mitochondrial velocity, we measured the angle of motile mitochondria on the kymographs using ImageJ and then calculated the velocity according to the following equation: “Velocity (µm/sec) = Tangent (Angle°+90°) * 0.111276”. Because the angles measured by ImageJ were all between 0°∼−180°, the values of “Tangent (Angle°)” were all under 0. Therefore, we added 90° to the measured angles to distinguish the anterograde and retrograde mitochondrial movements as positive and negative values, respectively. In our imaging system, 0.111276 represents the length of one pixel (µm). Mitochondrial motility was tracked by hand using kymographs and the cut-off value of 0.0019 µm/sec (mean velocity) was used to distinguish motile from stationary mitochondria. Quantitative analysis of mitochondrial length was also based on manual tracking using ImageJ on the first picture of the live cell images.

### Western blot analysis

To extract brain tissues, mice were anesthetized and perfused transcardially with phosphate-buffered saline (PBS). Cultured primary hippocampal neurons in 6 well plates were harvested as cell pellets. Brain tissues and harvested cell pellets were resuspended in RIPA buffer (150 mM NaCl, 1% Nonidet P-40, 0.5% deoxycholic acid, 0.1% SDS, and 50 mM Tris-HCl, pH 7.4) containing protease inhibitors (Sigma, St. Louis, MO), Phenylmethylsulfonyl fluoride (PMSF; Sigma, St. Louis, MO), phosphatase inhibitors (A.G. Scientific, Inc., San Diego, CA) and TBA and then incubated on ice for 20 min and sonicated for 5 sec. After centrifugation at 18,000 g for 10 min, the supernatant was collected to remove membrane lipids. Protein concentrations were determined by BCA assay (Sigma), and equal amounts of protein were loaded on 10% glycine gels. The separated samples were transferred to a PVDF membrane and incubated with antibodies against the target proteins. Protein bands were visualized by enhanced chemilumin-escence (ECL; Amersham Pharmacia Biotech, Buckinghamshire, England) using a bioimaging analyzer (LAS-3000; Fuji, Tokyo, Japan). This process has been also described in a previous report [44].

### Immunocytochemistry analysis

Primary hippocampal neurons were plated on 18-mm round coverslips coated with poly-D-lysine which were in a 12-well plate. After Aβ and/or TBA treated, cells were washed with chilled PBS, and fixed with 4% paraformaldehyde (BIOSESANG, Inc., Gyeonggi-do, Korea) for 20 min at room temperature (RT). Cells were permeabilized and blocked with 1% Triton X-100 (Sigma-Aldrich, St. Louis, MO) and 1% goat serum (Vector Laboratories, Inc., Burlingame, CA) diluted in PBS for 10 min at RT. To detect acetylated α-tubulin, cells were incubated with primary antibodies (Sigma-Aldrich, St. Louis, MO) overnight at 4°C and then with secondary antibodies for 1 hr at RT. Primary and secondary antibodies diluted in permeabilizing and blocking solution. DAPI (0.1 µg/ml; Sigma-Aldrich, St. Louis, MO) staining was performed for 10 min at room temperature. Finally, coverslips were mounted on slides using mounting reagent (Biomeda corp., Foster city, CA). Cells were washed by PBS twice every steps between incubations. Images were acquired using confocal microscope (Olympus FV10i; Olympus, Tokyo, Japan).

### Statistical analysis

Significant differences between groups were examined for statistical analysis using t-test or one-way analysis of variance (ANOVA) and Bonferroni post-tests. All data were expressed as means ± SEM (*P<0.05, **P<0.01, ***P<0.001).

## Supporting Information

Movie S1 **Mitochondrial movement of control neuron.**
(MP4)

Movie S2 **Mitochondrial movement of TBA treated neuron.**
(MP4)

Movie S3 **Mitochondrial movement of Aβ treated neuron.**
(MP4)

Movie S4 **Mitohcondrial movement of Aβ and TBA treated neuron.** Hippocampal neurons from rat (E18) were plated with densities of 6×10^4^ cells in the somal side of the microfluidic chamber. Cells were transfected with pDsRed2-Mito after 7 days of culture. Cells were treated with Aβ (2 µM) for 24 hrs, and then treated with TBA (5 µM) for 3 hrs. Images were acquired every 1 sec for 2 min at microgrooves.
(MP4)

File S1 **Supporting** **Materials and Methods**.
(DOC)

## References

[1] Janke C, Kneussel M (2010) Tubulin post-translational modifications: encoding functions on the neuronal microtubule cytoskeleton. Trends Neurosci 33: 362–372. 20541813 10.1016/j.tins.2010.05.001

[2] Fukushima N, Furuta D, Hidaka Y, Moriyama R, Tsujiuchi T (2009) Post-translational modifications of tubulin in the nervous system. J Neurochem 109: 683–693. 19250341 10.1111/j.1471-4159.2009.06013.x

[3] Hammond JW, Cai D, Verhey KJ (2008) Tubulin modifications and their cellular functions. Curr Opin Cell Biol 20: 71–76. 18226514 10.1016/j.ceb.2007.11.010 PMC2274889

[4] Dent EW, Gertler FB (2003) Cytoskeletal dynamics and transport in growth cone motility and axon guidance. Neuron 40: 209–227. 14556705 10.1016/s0896-6273(03)00633-0

[5] Reed NA, Cai D, Blasius TL, Jih GT, Meyhofer E, et al (2006) Microtubule acetylation promotes kinesin-1 binding and transport. Curr Biol 16: 2166–2172. 17084703 10.1016/j.cub.2006.09.014

[6] Bulinski JC (2007) Microtubule modification: acetylation speeds anterograde traffic flow. Curr Biol 17: R18–20. 17208171 10.1016/j.cub.2006.11.036

[7] Zhang Y, Gilquin B, Khochbin S, Matthias P (2006) Two catalytic domains are required for protein deacetylation. J Biol Chem 281: 2401–2404. 16272578 10.1074/jbc.C500241200

[8] Stokin GB, Lillo C, Falzone TL, Brusch RG, Rockenstein E, et al (2005) Axonopathy and transport deficits early in the pathogenesis of Alzheimer's disease. Science 307: 1282–1288. 15731448 10.1126/science.1105681

[9] Hempen B, Brion JP (1996) Reduction of acetylated alpha-tubulin immunoreactivity in neurofibrillary tangle-bearing neurons in Alzheimer's disease. J Neuropathol Exp Neurol 55: 964–972. 8800092 10.1097/00005072-199609000-00003

[10] Henriques AG, Vieira SI, da Cruz ESEF, da Cruz ESOA (2010) Abeta promotes Alzheimer's disease-like cytoskeleton abnormalities with consequences to APP processing in neurons. J Neurochem 113: 761–771. 20345756 10.1111/j.1471-4159.2010.06643.x

[11] Ding H, Dolan PJ, Johnson GV (2008) Histone deacetylase 6 interacts with the microtubule-associated protein tau. J Neurochem 106: 2119–2130. 18636984 10.1111/j.1471-4159.2008.05564.x PMC2574575

[12] Calkins MJ, Reddy PH (2011) Amyloid beta impairs mitochondrial anterograde transport and degenerates synapses in Alzheimer's disease neurons. Biochim Biophys Acta 1812: 507–513. 21241801 10.1016/j.bbadis.2011.01.007 PMC3042500

[13] Decker H, Lo KY, Unger SM, Ferreira ST, Silverman MA (2010) Amyloid-beta peptide oligomers disrupt axonal transport through an NMDA receptor-dependent mechanism that is mediated by glycogen synthase kinase 3beta in primary cultured hippocampal neurons. J Neurosci 30: 9166–9171. 20610750 10.1523/JNEUROSCI.1074-10.2010 PMC6632489

[14] Rui Y, Tiwari P, Xie Z, Zheng JQ (2006) Acute impairment of mitochondrial trafficking by beta-amyloid peptides in hippocampal neurons. J Neurosci 26: 10480–10487. 17035532 10.1523/JNEUROSCI.3231-06.2006 PMC6674697

[15] Du H, Guo L, Yan S, Sosunov AA, McKhann GM, et al (2010) Early deficits in synaptic mitochondria in an Alzheimer's disease mouse model. Proc Natl Acad Sci U S A 107: 18670–18675. 20937894 10.1073/pnas.1006586107 PMC2972922

[16] Hirai K, Aliev G, Nunomura A, Fujioka H, Russell RL, et al (2001) Mitochondrial abnormalities in Alzheimer's disease. J Neurosci 21: 3017–3023. 11312286 10.1523/JNEUROSCI.21-09-03017.2001 PMC6762571

[17] Rice SE, Gelfand VI (2006) Paradigm lost: milton connects kinesin heavy chain to miro on mitochondria. J Cell Biol 173: 459–461. 16717123 10.1083/jcb.200604071 PMC2063855

[18] Frederick RL, Shaw JM (2007) Moving mitochondria: establishing distribution of an essential organelle. Traffic 8: 1668–1675. 17944806 10.1111/j.1600-0854.2007.00644.x PMC3739988

[19] Hubbert C, Guardiola A, Shao R, Kawaguchi Y, Ito A, et al (2002) HDAC6 is a microtubule-associated deacetylase. Nature 417: 455–458. 12024216 10.1038/417455a

[20] Zhang Y, Li N, Caron C, Matthias G, Hess D, et al (2003) HDAC-6 interacts with and deacetylates tubulin and microtubules in vivo. EMBO J 22: 1168–1179. 12606581 10.1093/emboj/cdg115 PMC150348

[21] Butler KV, Kalin J, Brochier C, Vistoli G, Langley B, et al (2010) Rational design and simple chemistry yield a superior, neuroprotective HDAC6 inhibitor, tubastatin A. J Am Chem Soc 132: 10842–10846. 20614936 10.1021/ja102758v PMC2916045

[22] Silva DF, Esteves AR, Arduino DM, Oliveira CR, Cardoso SM (2011) Amyloid-beta-Induced Mitochondrial Dysfunction Impairs the Autophagic Lysosomal Pathway in a Tubulin Dependent Pathway. J Alzheimers Dis 26 3:565–581. 21694451 10.3233/JAD-2011-110423

[23] Varadi A, Johnson-Cadwell LI, Cirulli V, Yoon Y, Allan VJ, et al (2004) Cytoplasmic dynein regulates the subcellular distribution of mitochondria by controlling the recruitment of the fission factor dynamin-related protein-1. J Cell Sci 117: 4389–4400. 15304525 10.1242/jcs.01299

[24] Anesti V, Scorrano L (2006) The relationship between mitochondrial shape and function and the cytoskeleton. Biochim Biophys Acta 1757: 692–699. 16729962 10.1016/j.bbabio.2006.04.013

[25] Lee JS, Yoon YG, Yoo SH, Jeong NY, Jeong SH, et al (2011) Histone deacetylase inhibitors induce mitochondrial elongation. J Cell Physiol 227 7:2856–2869. 10.1002/jcp.23027 21928346

[26] Caspersen C, Wang N, Yao J, Sosunov A, Chen X, et al (2005) Mitochondrial Abeta: a potential focal point for neuronal metabolic dysfunction in Alzheimer's disease. FASEB J 19: 2040–2041. 16210396 10.1096/fj.05-3735fje

[27] Morfini GA, Burns M, Binder LI, Kanaan NM, LaPointe N, et al (2009) Axonal transport defects in neurodegenerative diseases. J Neurosci 29: 12776–12786. 19828789 10.1523/JNEUROSCI.3463-09.2009 PMC2801051

[28] Chang DT, Reynolds IJ (2006) Mitochondrial trafficking and morphology in healthy and injured neurons. Prog Neurobiol 80: 241–268. 17188795 10.1016/j.pneurobio.2006.09.003

[29] Wang X, Su B, Siedlak SL, Moreira PI, Fujioka H, et al (2008) Amyloid-beta overproduction causes abnormal mitochondrial dynamics via differential modulation of mitochondrial fission/fusion proteins. Proc Natl Acad Sci U S A 105: 19318–19323. 19050078 10.1073/pnas.0804871105 PMC2614759

[30] Lei P, Ayton S, Bush AI, Adlard PA (2011) GSK-3 in Neurodegenerative Diseases. Int J Alzheimers Dis 2011: 189246. 21629738 10.4061/2011/189246 PMC3100544

[31] Chen S, Owens GC, Makarenkova H, Edelman DB (2010) HDAC6 regulates mitochondrial transport in hippocampal neurons. PLoS One 5: e10848. 20520769 10.1371/journal.pone.0010848 PMC2877100

[32] d'Ydewalle C, Krishnan J, Chiheb DM, Van Damme P, Irobi J, et al (2011) HDAC6 inhibitors reverse axonal loss in a mouse model of mutant HSPB1-induced Charcot-Marie-Tooth disease. Nat Med 17: 968–974. 21785432 10.1038/nm.2396

[33] Zilberman Y, Ballestrem C, Carramusa L, Mazitschek R, Khochbin S, et al (2009) Regulation of microtubule dynamics by inhibition of the tubulin deacetylase HDAC6. J Cell Sci 122: 3531–3541. 19737819 10.1242/jcs.046813

[34] Goldstein LS, Yang Z (2000) Microtubule-based transport systems in neurons: the roles of kinesins and dyneins. Annu Rev Neurosci 23: 39–71. 10845058 10.1146/annurev.neuro.23.1.39

[35] Dompierre JP, Godin JD, Charrin BC, Cordelieres FP, King SJ, et al (2007) Histone deacetylase 6 inhibition compensates for the transport deficit in Huntington's disease by increasing tubulin acetylation. J Neurosci 27: 3571–3583. 17392473 10.1523/JNEUROSCI.0037-07.2007 PMC6672116

[36] Wang X, Perry G, Smith MA, Zhu X (2010) Amyloid-beta-derived diffusible ligands cause impaired axonal transport of mitochondria in neurons. Neurodegener Dis 7: 56–59. 20160460 10.1159/000283484 PMC2859232

[37] Reddy PH (2007) Mitochondrial dysfunction in aging and Alzheimer's disease: strategies to protect neurons. Antioxid Redox Signal 9: 1647–1658. 17696767 10.1089/ars.2007.1754

[38] Mattson MP, Gleichmann M, Cheng A (2008) Mitochondria in neuroplasticity and neurological disorders. Neuron 60: 748–766. 19081372 10.1016/j.neuron.2008.10.010 PMC2692277

[39] Misko A, Jiang S, Wegorzewska I, Milbrandt J, Baloh RH (2010) Mitofusin 2 is necessary for transport of axonal mitochondria and interacts with the Miro/Milton complex. J Neurosci 30: 4232–4240. 20335458 10.1523/JNEUROSCI.6248-09.2010 PMC2852190

[40] Cha MY, Han SH, Son SM, Hong HS, Choi YJ, et al (2012) Mitochondria-specific accumulation of amyloid beta induces mitochondrial dysfunction leading to apoptotic cell death. PLoS One 7: e34929. 22514691 10.1371/journal.pone.0034929 PMC3325919

[41] Miller KE, Sheetz MP (2004) Axonal mitochondrial transport and potential are correlated. J Cell Sci 117: 2791–2804. 15150321 10.1242/jcs.01130

[42] Kaech S, Banker G (2006) Culturing hippocampal neurons. Nat Protoc 1: 2406–2415. 17406484 10.1038/nprot.2006.356

[43] Park JW, Vahidi B, Taylor AM, Rhee SW, Jeon NL (2006) Microfluidic culture platform for neuroscience research. Nat Protoc 1: 2128–2136. 17487204 10.1038/nprot.2006.316

[44] Boo JH, Song H, Kim JE, Kang DE, Mook-Jung I (2009) Accumulation of phosphorylated beta-catenin enhances ROS-induced cell death in presenilin-deficient cells. PLoS One 4: e4172. 19137062 10.1371/journal.pone.0004172 PMC2613523

