# Supplementary material for: HDAC6 Inhibitor Blocks Amyloid Beta-Induced Impairment of Mitochondrial Transport in Hippocampal Neurons
Source: PLoS One. 2012 Aug 22;7(8):e42983. doi: 10.1371/journal.pone.0042983 (PMC3425572; doi:10.1371/journal.pone.0042983)
Supplement: File S1 — Supporting Materials and Methods . (DOC) [file pone.0042983.s005.doc]

**HDAC6 Inhibitor Blocks Amyloid beta-induced Impairment of Mitochondrial Transport in Hippocampal Neurons**

**Supporting Information File S1**

Chaeyoung Kim,^1*^ Heesun Choi,^1*^ Eun Sun Jung,^1^ Wonik Lee,^1^ Soojung Oh,^2,3^ Noo Li Jeon,^2,3^ and Inhee Mook-Jung^1#^

^1^Department of Biochemistry and Biomedical Sciences, Seoul National University College of Medicine, Seoul, Korea, ^2^School of Mechanical and Aerospace Engineering, Seoul National University, Seoul, Korea, ^3^World class University (WCU) Program of Multiscale design, School of Mechanical and Aerospace Engineering, Seoul National University, Seoul, Korea

^#^Corresponding Author

Inhee Mook-Jung, PhD

Department of Biochemistry and Biomedical Sciences,

Seoul National University College of Medicine,

28 Yeongon-dong, Jongro-gu, Seoul 110-799, Korea,

Tel: +82-2-740-8245, Fax: +82-3672-7352

E-mail: inhee@snu.ac.kr

* These authors contributed equally to this work

Abbreviated title: Role of HDAC6 inhibitor in mitochondrial transport


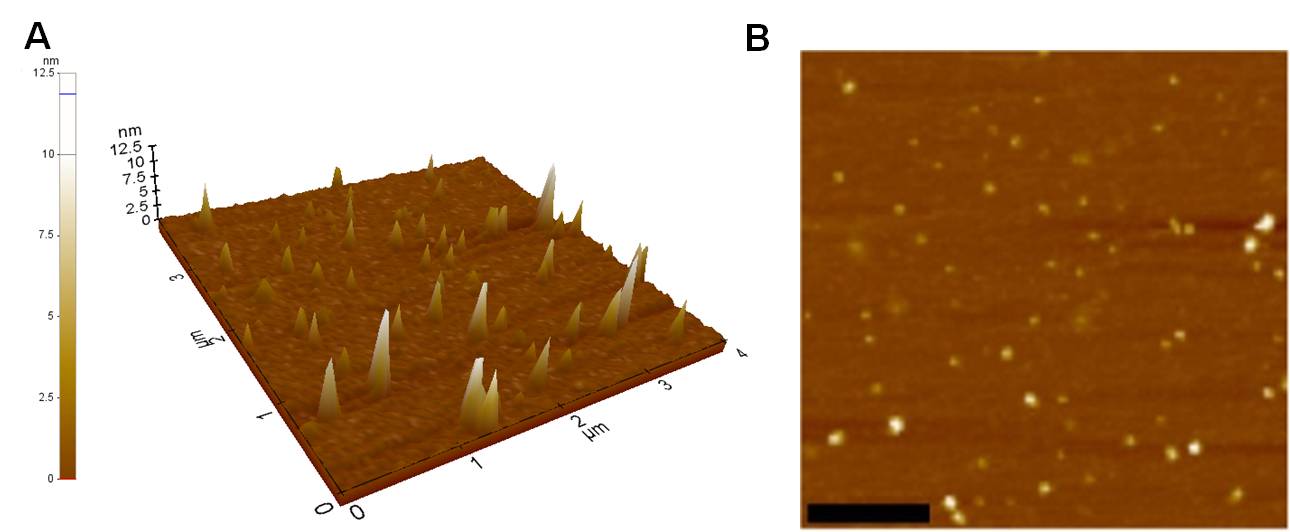


**Figure S1**. Atomic force microscopy 3D (A) and 2D (B) images of Aβ_1-42_. 2 μM Aβ was incubated in Neurobasal medium at 37 ℃ for 24 hr. The scale bar represents 1μm.

**Supporting Materials and Methods**

**Atomic Force Microscopy (AFM)**

Aβ_1-42_ peptides were incubated in Neurobasal medium at 37 ℃ for 24 hr for AFM analysis (PARK Systems Inc., Suwon, South Korea). 10 ml of Aβ (2 μM) spotted on freshly cleaved micas. The micas were dried at room temperature, and rinsed with distilled water. AFM images were acquired using Non-Contact AFM mode. Image data were acquired at scan rates at 0.5 Hz and analyzed by XEI analysis program Version 1.7.2 (PARK Systems Inc., Suwon, South Korea).
